# Thyroid dysfunction in infants with severe intestinal insufficiency: a case series

**DOI:** 10.1590/1984-0462/2023/41/2021402

**Published:** 2023-03-13

**Authors:** Gabriela Ibrahim Martins de Castro, Mário Cícero Falcão, Juliana Zoboli Del Bigio, Werther Brunow de Carvalho

**Affiliations:** a Universidade de São Paulo, São Paulo, SP, Brazil.

**Keywords:** Short bowel syndrome, Thyroid diseases, Thyroid hormones, Infant, Síndrome do intestino curto, Doenças da glândula tireoide, Hormônios tireóideos, Lactente

## Abstract

**Objective:**

The aim of this study was to describe the status of thyroid function in infants with severe intestinal dysfunction.

**Case description:**

A retrospective study was conducted in a tertiary neonatal intensive care center, including newborns and infants with severe intestinal dysfunction, hospitalized between 2015 and 2020. From the medical records, the following data were collected: gestational age, birth weight, underlying pathology that led to intestinal dysfunction, hospital stay, presence of thyroid dysfunction, age from the onset of thyroid dysfunction, initial and maximum dose of levothyroxine replacement, and levothyroxine administration route and outcome. Seven children (0.76% of 914 hospitalizations) developed severe intestinal insufficiency: vanishing gastroschisis (42.9%), Berdon syndrome (28.5%), apple peel (14.3%), and OIES syndrome (14.3%) – omphalocele, exstrophy of cloaca, imperforate anus, and spina bifida. The mean gestational age was 33.3±1.6 weeks, the mean birth weight was 2,113.9±370.9 g, the median hospitalization was 420 days, and mortality was 42.9%. Of these seven cases, four (57.1%) presented thyroid dysfunction, evaluated by blood hormone dosages and the dose of levothyroxine replacement ranged from 25 to 100 μg/day, administered by gastric or rectal route.

**Comments:**

This series of cases draws attention to thyroid dysfunction (hypothyroidism) in children with severe intestinal insufficiency receiving exclusive parenteral nutrition for a prolonged period, whose etiology is iodine deficiency, because, in Brazil, micronutrient solutions added to parenteral nutrition do not contain iodine.

## RESUMO

**Objetivo:**

Descrever o *status* da função tireoidiana em lactentes com disfunção intestinal grave.

**Descrição do caso:**

Estudo retrospectivo, realizado em um centro de terapia intensiva neonatal de nível terciário, que incluiu recém-nascidos e lactentes com disfunção intestinal grave, internados entre 2015 e 2020. Dos prontuários foram obtidos: idade gestacional; peso de nascimento; patologia de base que levou à disfunção intestinal; tempo de internação; presença de disfunção tireoidiana; idade no início da disfunção tireoidiana; dose inicial e máxima da reposição de levotiroxina; via de administração da levotiroxina e desfecho. Sete crianças (0,76% do total de 914 internações) evoluíram com insuficiência intestinal grave: *vanishing* gastrosquise (42,9%), síndrome de Berdon (28,5), *apple peel* (14,3%) e síndrome onfalocele, ânus imperfurado, extrofia de cloaca e espinha bífida — OIES (14,3%). A média de idade gestacional foi de 33,3±1,6 semanas e de peso ao nascimento de 2113,9±370,9 gramas; a mediana de internação foi de 420 dias e a mortalidade foi de 42,9%. Desses sete casos, quatro (57,1%) apresentaram disfunção tireoidiana, avaliada por dosagens hormonais séricas, e a dose de reposição de levotiroxina variou de 25 a 100 mcg/dia, administrada por via gástrica ou retal.

**Comentários:**

Esta série de casos chama a atenção para a disfunção tireoidiana (hipotireoidismo) em crianças com insuficiência intestinal severa que receberam nutrição parenteral exclusiva por tempo prolongado, cuja etiologia é a deficiência de iodo, pois no Brasil as soluções de micronutrientes adicionadas à nutrição parenteral não contêm iodo.

## INTRODUCTION

In the neonatal period, hypothyroidism occurs due to embryological defects in the development of the thyroid or in the synthesis of its hormones. Other probable causes are transport defects or hormonal action, central hypothyroidism, iodine deficiency, and transient hypothyroidism (iodine excess, exposure to antithyroid drugs, thyroid hemangiomas, DUOX2 gene mutation, and maternal antithyroid antibodies).^1^

Thus, situations in which prolonged periods of fasting occur, and the use of prolonged parenteral nutrition is mandatory, newborns and infants experience a deprivation of iodine supply, because, in Brazil, parenteral nutrition solutions do not contain this nutrient and may evolve with thyroid dysfunction due to deficit of hormonal production.^2^

Conditions that may evolve with hypothyroidism due to iodine deficiency are intestinal insufficiencies, defined by the absence of sufficient intestine to meet nutritional and metabolic needs for the growth and development of the infant.^2^

Short bowel syndrome is the main cause of severe intestinal failure in newborns and infants.^3^ The main causes of short bowel syndrome in children are massive resections secondary to necrotizing enterocolitis, gastroschisis (simple, complex, and “vanishing”), intestinal atresia and malrotation associated with intestinal volvulus, and necrotizing enterocolitis accounts for more than 90% of cases.^4^

Other situations leading to severe intestinal insufficiency are Berdon syndrome (microcolon with intestinal hypoperistalsis and megabladder)^5^ and “apple peel syndrome” (small bowel atresia caused by occlusion of the superior mesenteric artery, causing a winding of the small intestine around the spiral vascular axis).^6^

Intestinal insufficiency causes several metabolic changes in the organism, associated with poor growth, intestinal adaptation, and bone metabolic disease; however, there is less information in the literature regarding endocrine functions, such as the pituitary hypothalamus axis and particularly on thyroid function in children with severe intestinal insufficiency.^2^

## CASE REPORT

This study was conducted at the Neonatal Intensive Care Center-2 (CTIN-2), Instituto da Criança e do Adolescente do Hospital das Clínicas da Faculdade de Medicina da Universidade de São Paulo, São Paulo, Brazil, including newborns and infants with severe intestinal dysfunction who were hospitalized between 2015 and 2020.

The project was approved by the Ethics Committee of the Department of Pediatrics and the Research Project Analysis Committee of the Hospital das Clínicas (protocol no.: 4,709,755, CAAE: 45676621.5.0000.0068).

During 2015–2020, CTIN-2 had 914 hospitalizations (clinical and surgical). Of these, seven children (0.76%) have evolved with severe intestinal insufficiency.

The analysis of these seven cases showed mean gestational age (in weeks) at birth of 33.3±1.6 and mean weight (in grams) at birth of 2113.9±370.9.

Regarding the pathology that led to severe intestinal insufficiency, the following were observed: vanishing gastroschisis (42.9%), Berdon syndrome (28.5), apple peel (14.3%), and OIES syndrome (14.3%) – acronym of a malformations complex association including omphalocele, exstrophy of bladder or cloaca, imperforate anus, and spinal defects. Among other relevant pathologies, cholestasis (defined as direct bilirubin greater than 2 mg/dL) was observed in 42.9% of the cases. The median of hospitalization days was 420 (37–690) and the mortality was 42.9%. It is noteworthy that among these children with severe intestinal insufficiency, 5/7 (71.4%) had short bowel syndrome (loss of more than 80% of small intestine and cecal ileus valve).

Table 1 shows gestational age, birth weight, underlying pathology that led to severe intestinal insufficiency, other relevant pathologies not related to the underlying disease, hospitalization time in CTIN-2, and outcome (discharge, death, or transfer) of the seven selected cases.

**Table 1.** Gestational age (weeks), birth weight (grams), underlying pathology, other pathologies, hospital stay (days), and outcome

	GA (weeks)	Birth weight (g)	Basic disease	Other diseases	LOS (days)	Outcome
Case 1	34.7	1,640	Multiple intestinal atresia (Apple-peel)	Biliary hamartoma	346	Transfer
Case 2	34.4	2,675	Vanishing gastroschisis	–	37	Death
Case 3	33.7	1,960	Vanishing gastroschisis	–	242	Transfer
Case 4	30.4	1,700	OIES	Hepatic thrombosis	690	Transfer
Case 5	34.4	2,290	Vanishing gastroschisis	Cholestasis	678	Transfer
Case 6	31.7	2,180	Berdon syndrome	Cholestasis	600	Death
Case 7	33.8	2,350	Berdon syndrome	Cholestasis	420	Death

GA: gestational age in weeks; LOS: length of hospital stay in days; OIES: association of omphalocele, imperforate anus, exstrophy of cloaca, and spina bifida; Berdon syndrome: Megacystis-microcolon-intestinal hypoperistalsis syndrome; Transfer: transfer from Neonatal Intensive Care Unit to Pediatric Ward.

In CTIN-2, children with prolonged fasting and total parenteral nutrition, thyroid dysfunction screening is routine. The original protocol, created in 2015, established monthly dosage of free thyroxine (Free T4) and thyroid stimulator hormone (TSH). In 2021, the service adopted a routine, which includes serum determination of antithyroid antibodies to exclude thyroiditis, and ultrasound of the thyroid gland to detect nodules (blood and urinary iodine dosages are not available in the unit). Thus, for the seven cases described in this publication, this new protocol was not applied.

Table 2 shows the laboratory evolution in relation to the minimum values of Free T4 and maximum TSH in the seven cases studied.

**Table 2.** Minimum values of free T4 and maximum values of thyroid stimulator hormone.

	Minimum values of free T4 (ng/dL)	Maximums values of TSH (mIU/mL)
Case 1	1.75	2.60
Case 2	0.42	110.30
Case 3	0.70	5.50
Case 4	0.96	4.03
Case 5	1.00	4.64
Case 6	0.45	169.10
Case 7	0.87	60.85

Normal values: free T4: 0.93–1.70 ng/dl (electrochemoimmunoassay); TSH: thyroid stimulator hormone 0.27–4.2 mIU/ml (electrochemiluminoluminometric).

The analysis of the values in Table 2 shows that cases 2, 3, 6, and 7 had altered hormone determination (Free T4 and TSH) and were considered to have thyroid dysfunction (hypothyroidism). Cases 1 and 4 presented normal values of these hormones, therefore, without thyroid dysfunction; case 5 presented slightly altered TSH, which was not confirmed in a new determination, thus excluding hypothyroidism. Therefore, of the seven evaluated cases, 4/7 showed thyroid dysfunction, totaling 57.1%.

Table 3 shows the age in the beginning of thyroid dysfunction, the initial dose of levothyroxine supplementation (μg/kg), the therapeutic dose of the same drug (μg/kg), and administration route (gastric or rectal route) in the seven selected cases. The application technique of levothyroxine rectally followed the steps: the drug is portioned by the pharmacy in the prescribed dose, dilution is performed in the unit with 2–3 mL of distilled water (depending on the dose of levothyroxine), the rectal tube used is number 8, this tube is introduced 2–3 cm (according to the child’s weight, less than 2,000 g, 2 cm), and then the administration of the tube is washed with 1 ml of distilled water and then removed.

**Table 3.** Age of onset of thyroid dysfunction (days of life), initial dose of levothyroxine supplementation (μg), therapeutic dose of levothyroxine (μg), and administration route.

	Age of onset of thyroid dysfunction (days of life)	Initial dose of levothyroxine (μg)	Levothyroxine therapeutic dose (μg)	Levothyroxine administration route
Case 1	No dysfunction	–	–	–
Case 2	114	25	50	Gastric
Case 3	86	25	25	Gastric
Case 4	No dysfunction	–	–	–
Case 5	No dysfunction	–	–	–
Case 6	34	50	100	Rectal
Case 7	27	25	50	Rectal

The routine in CTIN-2 for levothyroxine replacement in cases of hypothyroidism is to start with doses between 12.5 and 25 μg, with subsequent dosage of Free T4 and TSH, in the interval between 15 and 30 days after the start of treatment and, if necessary, the dose is increased. Thyroid function of these children was normalized after 15–30 days of levothyroxine replacement with effective doses, and no differences were found between gastric and rectal route. With the therapeutic doses listed in Table 3, all four children showed levels within the normal range of the hormones collected. It is noteworthy that none of these children had alterations in the neonatal screening test for hypothyroidism.

## DISCUSSION

The literature points to some publications on the pituitary hypothalamus axis in some situations such as sepsis, shock, and trauma in the pediatric group.^7^ However, these publications practically address the pituitary hypothalamus axis adrenal, with detailed explanations about adrenal dysfunction in critically ill children.^8^ In contrast, few reports point to changes in the pituitary hypothalamus axis in other endocrine glands. It is also emphasized that there are no experimental studies relating intestinal failure and thyroid pituitary hypothalamus axis.^2^

The thyroid pituitary hypothalamus axis begins in fetal life and is dependent, in the first months of pregnancy of maternal hormones. In the second trimester of pregnancy, the fetal thyroid begins to produce hormones and, after birth, there is a substantial increase in the production of triiodothyronine and thyrotoxin. Preterm infants, due to hypothalamic axis immaturity, have lower levels of thyrotoxin, as well as children with severe diseases may present with thyroid dysfunction, even with normal thyroid function prior to the disease.^9^ These situations are called nonthyroidal illness syndrome.^2^ The situation of the thyroid pituitary hypothalamus axis has practically unstudied in children with intestinal failure who were dependent on exclusive parenteral nutrition. In addition, it is assumed that preterm infants with short bowel syndrome, a noninfrequent association, evolve with more pronounced hypothyroidism.

Iodine is an essential trace element for the synthesis of thyroid hormones, responsible for brain development, neuron proliferation, and regulation of processes involving brain functions, and iodine deficiency is the first cause of preventable mental deficit.^10^

Infants with exclusive parenteral nutritional support for prolonged periods are at risk of multiple nutrient deficiencies. Deficiencies in iron, zinc, vitamins, and magnesium are already well known, but little is known about the status of iodine and its consequences in children with prolonged parenteral nutrition.^10^ In this brief report of seven cases, the probable cause of thyroid dysfunction in four of them was iodine deficiency, since all had normal thyroid function in the neonatal screening and received total parenteral nutrition without iodine.

In a study conducted in Norway in 2020,^11^ micronutrient status was evaluated in children with intestinal failure receiving home parenteral nutrition. Regarding iodine, although the children recovered this nutrient in parenteral nutrition (median 2.7 μg/kg/day), all children presented some degree of iodine deficiency, defined by ioduria below 100 μg/L. In addition, the authors pointed out that there are few reported cases of hypothyroidism in children receiving exclusive parenteral nutrition containing iodine, in contrast to parenteral nutrition without iodine, where the risk of developing a thyroid dysfunction is 33%,^11^ a rate much lower than that found in this study (57.1%). It is also worth discussing the fact that it does not form all children with severe intestinal dysfunction, fasting, and parenteral nutrition without iodine replacement who evolved with thyroid dysfunction (57.1%). A plausible explanation for this may be a greater maternal iodine reserve, with greater passage of this micronutrient in fetal life and consequently higher iodine status in the newborn, conferring a certain protection in thyroid function.

Thus, screening for thyroid dysfunction should be mandatory in children with prolonged fasting receiving exclusive parenteral nutrition without iodine replacement.

The European Society for Paediatric Gastroenterology Hepatology and Nutrition (ESPGHAN) suggests that children with prolonged parenteral nutrition should be monitored for the iodine status (blood sample and urine 24-h sample dosages) and screened for hypothyroidism (Free T4 and TSH) periodically, without specifying the time interval between collections.^12^

The analysis of the cases studied showed a mean gestational age of 33.3 weeks and birth weight of 2113.9 g, all of which were premature (gestational age less than 37 weeks) and birth weight of 6/7 (85.7%) was less than 2,500 g. Premature children have an elevated risk of micronutrient deficiency, including iodine.

Some considerations should be emphasized in relation to the recommended doses on iodine supplementation in children receiving exclusive parenteral nutrition.

The American Society of Clinical Nutrition since 1988^13^ recommends the dose of 1 μg/kg/day of iodine in parenteral nutrition to avoid deficiency of this nutrient; the same is the recommendation of ESPGHAN (2005).^14^

In contrast, the recommendations published in 2018 determine that preterm infants should receive in parenteral nutrition iodine in doses ranging from 1 to 10 μg/kg/day and infants and children older, at least 1 μg/kg/day (strong recommendation).^12^ In this same publication, the authors show that there are discrepancies in the doses of iodine supplementation in parenteral nutrition (10–30 μg/kg/day).^15,16^

Thus, ideally serum and urinary determination of iodine should be a routine laboratory test in cases of intestinal insufficiency, fasting, and prolonged parenteral nutrition. Plasma iodine measurement is performed by atomic mass spectrometry (expensive and complex method) in a sample of 0.5 ml of plasma and normal values are between 52 and 109 μg/l. Urinary iodine can be measured in an isolated sample, a minimum volume of 3 ml, using indirect detection by the Sandell-Kolthoff reaction. Urinary determination in a 24 h sample is more reliable; however, this method presents the inconvenience of prolonged collection and the need for refrigeration. The method used is mass spectrometry, like the plasma determination. Values below 25 μg/l indicate severe iodine deficiency.^17^ The service where this study was conducted does not have these laboratory methods.

Another theme that deserves reflection is the route of administration of levothyroxine, because there is only a presentation for oral/enteral route and children with severe intestinal dysfunction usually have large volumes of gastric drainage and need to remain with the gastric tube open.

The simplest and most physiological way to administer the hormone would be the gastric route (by naso or orogastric tube), with closure after, for at least 1 h. However, some children do not tolerate this closure, presenting vomiting and gastric distension. In addition, it is difficult to know whether levothyroxine will be absorbed, because there is very little absorption surface in short bowel syndrome. Even so, in two children in this study, we opted for the rectal route for hormone replacement, with normalization of thyroid function. The Instituto da Criança e do Adolescente has two publications, both from 2018,^2,18^ using the rectal route and resolution of thyroid dysfunction, justifying the hormonal replacement of two of the seven children in this study by the rectal route and reversal of hypothyroidism. No impact on levothyroxine supplementation was observed on growth (weight, length, and head circumference) or on enteral nutrition establishment.

The last topic of this discussion is about the age of onset of thyroid dysfunction, which ranged from 27 to 114 days, very different from the previous report of the Instituto da Criança e do Adolescente,^2^ which showed the onset of the condition between 5 months and 12 years, but children who developed short bowel syndrome at an older age were included.

Finally, the proper functioning of the thyroid gland favors the process of intestinal adaptation, as demonstrated in experimental models of short bowel syndrome, where the replacement of thyroid hormones promotes enterocyte and intestinal villi proliferation.^19^

In conclusion, severe intestinal insufficiency, per se, seems to have no relation to the alteration of thyroid function. The development of hypothyroidism in these children is related to iodine deficiency, because in Brazil, as in many other countries, the parenteral nutrition solution does not include this nutrient and, in situations of prolonged fasting and total parenteral nutrition, iodine deficiency can lead to thyroid dysfunction.

Although, there is less information on the hypothalamus-pituitary-thyroid axis in children with severe intestinal insufficiency,^2^ this study showed that this theme deserves further research, especially in relation to the etiology of hypothyroidism due to iodine deficiency, that is, before the triggering of a nonthyroidal illness syndrome.

Finishing remains a great question to be answered. Once intestinal recovery occurs, that is, the provision of iodine can be made by diet; will the thyroid of these individuals return to normal function? Or will they be dependent on hormone replacement for the rest of their lives?
